# The Potential Cost of Cultural Fit: Frame Switching Undermines Perceptions of Authenticity in Western Contexts

**DOI:** 10.3389/fpsyg.2018.02622

**Published:** 2018-12-20

**Authors:** Alexandria L. West, Rui Zhang, Maya A. Yampolsky, Joni Y. Sasaki

**Affiliations:** ^1^Department of Psychology, York University, Toronto, ON, Canada; ^2^Department of Psychology, Dickinson College, Carlisle, PA, United States; ^3^Department of Psychology, Laval University, Quebec City, QC, Canada; ^4^Department of Psychology, University of Hawai’i at Mānoa, Honolulu, HI, United States

**Keywords:** frame switching, authenticity, cultural fit, bicultural, consistency, biculturalism, multiculturalism, multicultural experience

## Abstract

Behaving consistently across situations is fundamental to a person’s authenticity in Western societies. This can pose a problem for biculturals who often frame switch, or adapt their behavior across cultural contexts, as a way of maintaining fit with each of their cultures. In particular, the behavioral inconsistency entailed in frame switching may undermine biculturals’ sense of authenticity, as well as Westerners’ impressions of biculturals’ authenticity. Study 1 had a diverse sample of biculturals (*N* = 127) living in the United States and Canada describe an episode of frame switching (vs. no switching control vs. neutral control) and report on their state authenticity during the episode. Results showed that biculturals recalled feeling less authentic during an instance of frame switching compared to no switching control and neutral control. Study 2 had mainstream Canadians (White and of American, Canadian, or Western European cultural heritage, *N* = 97) read a hypothetical vignette, from a third-person perspective, about a bicultural who frame switches (vs. no switching control vs. neutral control) and provide their impressions of the bicultural’s authenticity and multiple other desirable traits. Participants rated the bicultural as less authentic when he frame switched compared to no switching control and neutral control, and rated him as less likeable, trustworthy, and warm (but not competent) as downstream consequences of seeing him as less authentic. These results demonstrate that frame switching can come at a cost to authenticity, both in terms of how biculturals see themselves and are seen by others, at least in Western societies. These findings highlight that the way biculturals negotiate their cultures affects them psychologically and socially. In the context of cultural fit, the active process of establishing and maintaining fit with one’s cultures can have unforeseen consequences.

## Introduction

“This above all: To thine own self be true, And it must follow, as the night the day, Thou canst not then be false to any man.” Shakespeare, Hamlet, 1.3.78–80.

Authenticity is a virtue, a quality we strive toward for ourselves and prize in those around us. It is most commonly defined as knowing and behaving according to our true selves (Rogers, 1961; Deci and Ryan, 1985; Barrett-Lennard, 1998; Harter, 2002; Wood et al., 2008). Resisting external influence can signal that our behavior reflects our true selves, at least in Western cultures, hence, one essential way that people in these cultures maintain authenticity is by behaving consistently across different situations with different people (Wood et al., 2008). Behaving consistently may be simple enough for people who mainly interact with relatively homogenous social groups but can prove problematic for those whose social groups are more distinct. Biculturals, who identify with at least two cultures, often adapt themselves to each of their cultural contexts—a process called frame switching (Hong et al., 2000). Frame switching enables biculturals to fit in with both of their cultural groups, which can benefit them in many ways (LaFromboise et al., 1993; Phinney and Devich-Navarro, 1997; David et al., 2009; Mistry and Wu, 2010). Yet, because adapting to distinct cultures often requires behaving inconsistently overall, it is possible that biculturals may experience certain costs in contexts where the mainstream culture highly values consistency. Here, we focus on Western contexts in which the mainstream culture is defined by the expectations, values, and beliefs held by White monoculturals descendant from Western Europe. The present research examines the consequences of biculturals’ behavioral inconsistency for their own sense of authenticity and others’ impressions of their authenticity in the United States and Canada.

### Frame Switching as a Cultural Fit Process

Cultural fit refers to the match between a person’s characteristics (e.g., traits, values, attitudes) and those of their cultural group (Searle and Ward, 1990; Ward and Chang, 1997). Past research has primarily examined cultural fit as a relatively stable, individual-level quality that people possess to different degrees, and it has focused on the outcomes associated with having more or less fit with a culture in general. Complementing this individual differences approach, we emphasize that cultural fit is also a dynamic psychological process through which people actively fit aspects of themselves (e.g., self-concept, emotions, behaviors, etc.) to the surrounding cultural context. In studying immigrants’ emotional cultural fit, for instance, findings on individual differences have highlighted the predictors of biculturals’ overall fit with their host and heritage cultures (De Leersnyder et al., 2011; De Leersnyder, 2017). However, in addition to a bicultural having the relatively stable ability to maintain a certain level of fit with both of their cultures, they can also dynamically shift their emotional patterns to fit each of their cultural groups (De Leersnyder, 2017). Thus, cultural fit is not only a static, global quality but also a process that results in changing levels of fit with each culture depending on the context. For biculturals, this dynamic aspect of cultural fit is analogous to frame switching, which involves adapting the way they think and behave to suit one of their culture’s norms and values at a time.

There is no single way biculturals negotiate their cultures. Biculturals use multiple strategies and vary in how much they employ different processes (LaFromboise et al., 1993; West et al., 2017), though most may be able to use each process to some extent. Frame switching is a commonly used process that involves activating one culture’s knowledge structures (i.e., cultural frame) in response to contextual cues (Hong et al., 1997, 2000; Hong and Khei, 2014). Through the process of frame switching, biculturals act as cultural chameleons who adapt the way they think and behave to meet the demands of the current cultural context. For instance, research has shown that Mexican American biculturals expressed their personalities differently depending on which language they were using (Ramírez-Esparza et al., 2006). When reporting on their traits in Spanish, their personality profiles were more similar to Mexican monoculturals than when they reported on their traits in English, presumably as a result of activating their Mexican cultural frame. The reverse also occurred, whereby their personality profiles were more similar to mainstream American monoculturals when they reported on their traits in English compared to Spanish, presumably because using English activated their American cultural frame. Replicating this demonstration of biculturals’ frame switching, Chen and Bond (2010) found that Hong Kong Chinese biculturals behaved differently when they were speaking to a mainstream American compared to a Hong Kong Chinese interviewer, manifesting traits that reflect the perceived personality prototypes for each culture (e.g., more extraverted for American, less open for Chinese). In other frame switching research, biculturals have been shown to adapt not only their personality and social behavior, but their values, emotions, and cognitive styles in response to cultural contextual cues (Ralston et al., 1995; Hong et al., 2000; Verkuyten and Pouliasi, 2002; Perunovic et al., 2007; Mok and Morris, 2009; Doucerain et al., 2013; Chen et al., 2014). Past researchers have generally considered frame switching an adaptive skill for biculturals because it helps them fulfill core human needs for competence and belonging with each of their cultural groups (LaFromboise et al., 1993; Phinney and Devich-Navarro, 1997; David et al., 2009; Mistry and Wu, 2010). Frame switching may indeed be an essential strategy for maintaining fit with multiple cultures, but might biculturals’ constant switching have consequences, particularly in cultural contexts that value consistency?

### Western Cultures Expect and Value Consistency

It is well established that people in Western cultures tend to dislike inconsistency. Research going back to classic investigations of cognitive dissonance, which were mostly based on observations of Americans, suggests that awareness of one’s inconsistencies can cause discomfort (Festinger, 1957; Elliot and Devine, 1994). We see everyday evidence of this in the condemnation of people who are “two-faced,” “flip-floppers,” or hypocrites. While Westerners are known to react negatively to many types of inconsistency (e.g., inconsistency between attitude and behavior), their reactions to inconsistency in behavior across contexts is most relevant in the case of frame switching. Western philosophical traditions broadly assume that unchanging, absolute truths form the basis of reality, in contrast to naïve dialectical assumptions of constant flux and contradictions (Peng and Nisbett, 1999; Spencer-Rodgers et al., 2010). This abstract assumption gives root to more explicit cultural beliefs underlying preferences for consistency. Specifically, the cultural aversion to behavioral inconsistency may be the product of two interrelated lay theories: dispositionism, which assumes that behavior is primarily caused by internal attributes, and an entity view of the self, which assumes that internal attributes are stable across situations and time (Chiu et al., 1997; Knowles et al., 2001). Together, these lay theories create a framework in which people in Western cultures expect themselves and others to behave consistently (Markus et al., 1997; English and Chen, 2011). In reality, people in all cultures are influenced by external forces and by internal attributes leading everyone to some degree of consistency as well as variability (e.g., Church, 2000; Fleeson, 2004). The point, however, is that these shared lay theories result in a cultural prescription for behavior in Western contexts: you *should* be consistent.

When consistency is expected, inconsistency can be costly. The effects of behavioral consistency have typically been studied by measuring how similarly a person enacts their traits with different people. Traditionally, researchers have used a cross-sectional, self-report approach to examine the consistency of the traits a person manifests across various social roles (e.g., friend, student, etc.; Sheldon et al., 1997; Suh, 2002; English and Chen, 2007; Church et al., 2008a; Boucher, 2011). Recent research using experience-sampling methods and statistical techniques that correct methodological confounds has challenged prior conclusions about the extent to which cultures differ in actual, as opposed to perceived, cross-role consistency (Church et al., 2008b, 2013; Locke et al., 2017) and whether actual consistency (vs. flexibility) is associated with greater well-being (Baird et al., 2006; Magee et al., 2018). Even though researchers are still investigating cross-cultural differences in actual behavioral consistency, many find self-reported differences in how consistent people perceive themselves to be. Importantly, these differences may reflect participants’ awareness of the desirability of consistent behavior in their respective cultures (Edwards, 1953) and their endorsement of overarching lay theories of behavior (Church et al., 2006, 2012). Relevant research has shown that although people in most cultures generally perceive themselves to be more consistent than inconsistent across roles, consistency is sometimes higher in non-dialectical cultures – for example, perceived cross-role consistency is higher in the United States versus Japan (Church et al., 2008a, 2012; Locke et al., 2017) and for European Americans versus Asian Americans (English and Chen, 2007, 2011). Other studies suggest that, at least when it comes to perceived behavioral consistency, there may be negative consequences for Westerners violating this culturally expected norm. Cross-role inconsistency, examined cross-sectionally, has been associated with lower psychological and subjective well-being (Donahue et al., 1993; Sheldon et al., 1997; Suh, 2002), worse relationship quality (English and Chen, 2011), and lower informant ratings of social skill and likeability (Suh, 2002). Other cross-sectional studies have found perceived cross-role inconsistency to be linked with lower adjustment outcomes (e.g., life satisfaction, affect, etc.) even in non-Western cultures, but the strongest negative relationships generally occur in more Westernized and less dialectical samples (Suh, 2002; Church et al., 2008a, 2014; Boucher, 2011).

Although actually varying one’s behavior may be a flexible, adaptive skill for people in general (Church, 2000; Fleeson, 2004), perceived violations of a culture’s prescribed level of behavioral consistency may still have negative effects. This presents a problem for biculturals in Western contexts who use frame switching as a primary way of negotiating their cultures. For a bicultural who identifies strongly with both of their cultures, the main goal of switching may be to align themselves to either of their cultural groups in order to feel like they belong and are accepted by both. Ironically, their attempts to make themselves consistent with each of their cultures may backfire because doing so requires them to be inconsistent *between* their cultures. If biculturals’ inconsistency is made salient, frame switching may create fallout for the way biculturals see themselves and are seen by others, particularly in a dominant cultural context that discourages inconsistency such as the United States and Canada.

### The Heart of the Problem: Inconsistency Can Signal Inauthenticity

A key factor in the potential negative effects of frame switching may be authenticity. The concept of authenticity has come to refer to several interrelated characteristics (e.g., genuineness, fidelity, credibility, sincerity, etc.) that are highly valued and sought in many spheres of life – we want to have authentic experiences, consume authentic products, be and be with authentic people (Handler, 1986; Cohen E., 1988; Wang, 1999; Lindholm, 2008; McCarthy, 2009; Sims, 2009; Grazian, 2010). The latter desire, which requires us to judge our own and others’ authenticity, is most relevant for our research and at its core rests on cultural expectations for what authenticity, or being true to oneself, should look like. Though people in all cultures experience authenticity (Slabu et al., 2014) and attempt to gauge others’ authenticity as a valuable social indicator (e.g., this person is a fraud, someone to trust), cultures differ in their understandings of what constitutes authentic behavior (Kanagawa et al., 2001; Kashima et al., 2004; Boucher, 2011; English and Chen, 2011; Kokkoris and Kühnen, 2014). We focus here on Western understandings of authenticity as a personal characteristic and its impact. Authenticity has long been considered a virtue in Western societies, and the writings of many philosophers, poets, and social scientists evidence its extensive intellectual tradition (Trilling, 1971; Handler, 1986; Harter, 2002; Kernis and Goldman, 2006; Braman, 2008; Lindholm, 2008). Over this time, scholars across and within disciplines have struggled to unanimously agree on the core features of authenticity. Some have focused on self-knowledge, or awareness of the true self, and others have focused on the importance of behavior, emphasizing that behavior must reflect and be directed by the true self (Rogers, 1961; Harter, 2002; Wood et al., 2008). The philosopher Jean Jacques Rousseau was a pivotal contributor to the Western understanding of authenticity and fervently argued that being authentic meant behaving only in line with one’s essence without regard for others’ opinions or inherently repressive social norms (Lindholm, 2008). On this point, the psychological literature has debated whether consistency and rejecting external influence are essential to authentic behavior. At times, research has treated cross-role consistency as a defining manifestation of authenticity (e.g., Block, 1961; Roberts and Donahue, 1994; Sheldon et al., 1997). Such research posits that variation between roles is caused by behavioral deviations from the true self in at least some of these roles (Deci and Ryan, 1985, 1991; Ryan, 1995; Sheldon et al., 1997), presumably due to external pressures rather than autonomous motivations (Wood et al., 2008). More recent investigations of the features of authentic and inauthentic states, however, suggests that people can still feel authentic even when accepting external influence (Slabu et al., 2014; Lenton et al., 2016). This debate highlights the potential dissociation between lay people’s (and even researchers’) actual experiences of authenticity and their beliefs about what authenticity should be.

Whereas scholars may still be exploring the nature of authenticity and debating the necessity of consistency to the construct for the purpose of research, the typical Western lay understanding of authenticity seems fundamentally at odds with behavioral inconsistency. Shakespeare’s famous quote, “To thine *own self* be true,” [emphasis added] is frequently cited by researchers and lay people alike for its defining embodiment of authenticity (e.g., Kernis and Goldman, 2006; Wood et al., 2008; Kifer et al., 2013). This prescription underscores the cultural expectation that people’s behavior should be expressive of their core self-understanding and that to do otherwise is to misrepresent oneself. Behavior which is inconsistent across situations, therefore, may be perceived negatively because inconsistent behavior can indicate that a person is being influenced by external factors rather than being their “true self” (Wild, 1965; Wood et al., 2008). Empirically, Kashima et al. (2004) found that Western participants in the United Kingdom, Australia, and Germany believed that a more context-sensitive self is less consistent and less of a true self, demonstrating their shared cultural associations between accepting external influence, inconsistency, and inauthenticity. This stands in contrast to certain Eastern cultures where people are believed to have malleable selves and are expected to adjust their behavior across roles (Markus and Kitayama, 1991, 1994, 1998), and doing so is not seen as inauthentic (English and Chen, 2011). For example, the same study (Kashima et al., 2004) found that Japanese participants believed that a more context-sensitive self, despite being less consistent, is more of a true self. As evidence of Westerners’ internalized understanding of authentic behavior, other studies show that Americans who see themselves as less consistent across social roles see themselves, and can be seen by others, as less authentic (Sheldon et al., 1997; Suh, 2002; Cross et al., 2003; English and Chen, 2011). Importantly, people’s judgments of their own and others’ authenticity based on behavior may draw more heavily on these shared cultural expectations of what authenticity *should* look like than how authentic behaviors actually feel in the moment. To illustrate, research on lay beliefs about authenticity in the United States suggests that Americans intuitively hold the dominant cultural belief that people should behave in line with their traits in order to be authentic (Fleeson and Wilt, 2010). For example, although introverts actually feel more authentic during moments in which their behavior is more extraverted, those who are asked to recall such an event remember feeling less authentic presumably because they believe that acting out of character reflects *in*authenticity, and this influences the way they reconstruct and interpret their experience (Fleeson and Wilt, 2010). Similarly, we posit that although adjusting one’s behavior to match a particular context may not feel inauthentic in the moment, reflecting on the inconsistency of one’s own or another’s behavior across contexts may negatively affect impressions of authenticity because of internalized Western associations between behavioral consistency and authenticity. This assertion may hold not only for mainstream members of Western cultures (i.e., White monoculturals of Western European cultural heritage), but also for biculturals living in these societies. Regardless of their heritage cultures, biculturals may still be affected by expectations to be consistent in the mainstream culture and they may at times judge themselves through this lens. Thus, frame switching in a Western context may negatively impact not only others’ impressions of whether a bicultural is authentic, but also the bicultural’s judgments about their own feelings of authenticity.

Diminished authenticity has a host of consequences. Previous studies of authenticity in Western contexts have shown that self-perceived inauthenticity predicts lower subjective and psychological well-being in terms of life satisfaction, role satisfaction, affect, self-esteem (e.g., Wood et al., 2008; Kifer et al., 2013), self-actualization, vitality, stress and coping (e.g., Kernis and Goldman, 2006), and anxiety and depression (e.g., Sheldon et al., 1997), among other negative outcomes. Other research points to the interpersonal consequences of and inauthenticity. For example, people who perceive their romantic partners as less authentic subsequently view them as less trustworthy, and are less committed to them (Wickham, 2013). Research on the authenticity of emotions shows that people feel less authentic when they hide their feelings, and this negatively affects their relationships in terms of satisfaction and social support (English and John, 2013) and their own and their partner’s emotional state, satisfaction, and commitment (Impett et al., 2012). These consequences are more pronounced for those who more strongly endorse the typically North American, independent self-construal (Le and Impett, 2013) or non-dialectical self-beliefs (Boucher, 2011). The social consequences of inauthenticity are thought to occur, at least in part, because inauthentic people can be seen as less honest, trustworthy, likeable, and socially competent (Reis and Patrick, 1996; Suh, 2002; Kernis and Goldman, 2006; Lopez and Rice, 2006; Krumhuber et al., 2007; Wickham, 2013). Taken together, these findings suggest that the cost of frame switching for North American biculturals may not stop at authenticity, but may have widespread downstream consequences as well. Specifically, the secondary predictions of the present research are that biculturals’ diminished authenticity due to frame switching will have subsequent costs to their subjective well-being and to the impressions people form of them on fundamental trait dimensions.

### Present Research Overview

The present research explores the complexity of maintaining cultural fit with multiple cultures, unveiling psychological and social consequences of biculturals’ frame switching. Although frame switching can enable cultural fit when a bicultural is in each frame, it may paradoxically undermine their fit with Western culture because the behavioral inconsistency involved in switching between frames violates cultural expectations and values (Markus et al., 1997; Sheldon et al., 1997; Suh, 2002; English and Chen, 2011). Thus, frame switching may come at a cost to biculturals’ authenticity in the United States and Canada, both in terms of how they see themselves (Study 1) and how they are seen by mainstream members of such societies (Study 2).

An overarching goal guiding our research is to understand the shared experiences of biculturals who may negotiate their cultures in similar ways despite the diversity of their specific backgrounds (West et al., 2017; Zhang et al., 2017). Thus, the present research is designed to capture the frame switching experiences of a diverse population of biculturals in a shared Western context. In Study 1, we sampled people living in Canada or the United States who identified as bicultural, regardless of their specific cultural backgrounds. Importantly, the manipulations for both Studies 1 and 2 target the effects of switching between cultural frames rather than the effects of specific cultural frames. In order to more broadly understand the experience and consequences of frame switching for American and Canadian biculturals, we examine both biculturals’ perception of their own past experiences via a recall task (Study 1) and mainstream members’ perceptions via a hypothetical vignette (Study 2).

## Study 1

Study 1 aimed to test whether frame switching makes American and Canadian biculturals feel less authentic, subsequently lowering their well-being. Bicultural participants recalled an experience of frame switching (vs. no switching control vs. neutral control) and reflected on how authentic they felt during the experience, followed by a report of their current sense of subjective well-being. We hypothesized, first, that frame switching would decrease state authenticity relative to the two control conditions. Second, we also hypothesized that frame switching, compared to either control condition, would negatively impact well-being via lower authenticity.

### Methods

#### Participants

One hundred and seventy-seven biculturals completed the study online for pay (1 GBP) on a crowdsourcing platform, Prolific Academic. Using prescreening items, eligibility criteria were that participants identified as multicultural (vs. monocultural)^1^, currently resided in the United States or Canada, and were fluent in English. Prior to any data analysis, we excluded participants who failed more than one of four attention checks (e.g., recall a term described on the previous page; select the “agree” response option for this item) or responded “No” to an item asking if they felt they completed the study honestly and attentively (*n* = 38). We also excluded participants from analysis if their responses on the manipulation task did not conform to the task’s instructions (*n* = 12). These exclusions resulted in a final sample of 127 participants^2^ (60 females, *M*_age_ = 30.70, *SD*_age_ = 10.41). The ethnic breakdown of this sample was approximately 37.8% White, 21.3% Mixed, 18.1% East Asian, 8.7% Black, 5.5% South Asian, 4.7% Latin American, 2.4% Native, and 1.6% Other.

#### Design and Procedure

After providing informed consent, all participants indicated the two cultures with which they most strongly identify and were then randomly assigned to one of three conditions of the recall manipulation: (1) Switching (*n* = 43), which emphasized behavioral inconsistency when frame switching, (2) No Switching control (*n* = 40), which emphasized behavioral consistency when actively not frame switching, or (3) Neutral Control (*n* = 44), which emphasized mundane behavioral inconsistency across different times of day. Finally, participants completed state authenticity, well-being (life satisfaction including social approval)^3^, and demographic measures, followed by debriefing.

#### Materials

##### Recall manipulation

Participants were instructed to spend 3–5 min writing about a past experience. In the Switching condition, participants wrote about a situation where they were with one of their cultural groups, and their behavior would have been different had they been with their other cultural group. In the No Switching condition, participants described a situation where they were with one cultural group, and their behavior would have been the same had they been with the other cultural group. In the Control condition, participants wrote about an instance of mundane switching: how they were different while completing their morning routine compared to their evening routine on an average day.

##### State authenticity

Lenton et al. (2013) measure of state authenticity was slightly reworded to ask about participants’ sense of authenticity during the situation they wrote about in the recall task rather than the present. The resulting 12-item measure (α = 0.90) assessed feelings and beliefs covering three defining factors of authenticity (Wood et al., 2008): *authentic living* (e.g., “I behaved in accordance with my values and beliefs”), *accepting external influence* (e.g., “I felt greatly influenced by other people,” reversed), and *self-alienation* (e.g., “I felt as if I didn’t know myself very well,” reversed). Participants reported their agreement with each statement on 7-point Likert scales from 1 (*strongly disagree*) to 7 (*strongly agree*).

##### Subjective well-being

*Satisfaction with life*. The 5-item Satisfaction with Life Scale (SWLS; α = 0.88) assesses how globally satisfied participants are with their lives (Diener et al., 1985) and has frequently been used to measure subjective well-being in previous work addressing similar research questions. Participants indicate their extent of agreement from 1 (*strongly disagree*) to 7 (*strongly agree*) with statements about their life satisfaction in terms of their own standards (e.g., “In most ways, my life is close to my ideal;” “So far I have gotten the important things I want in life”).

*Social approval*. We also added two items (α = 0.70) to the traditional Satisfaction with Life Scale to assess how much participants believe that important others approve of their lives: “I feel that I live up to the expectations of people close to me” and “People close to me approve of how I live my life” (Kim et al., 2008). We included these two items to be more inclusive of the cultural diversity of our sample, given that previous research suggests that social approval may be an important aspect of subjective well-being in many non-Western cultures (e.g., Suh, 2002).

### Results

**FIGURE 1:**
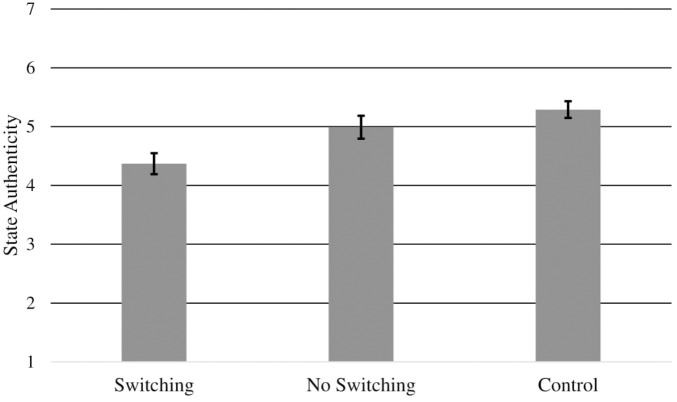
Study 1: Average state authenticity (±*SE*) by condition.

Addressing our first hypothesis on the effect of condition on state authenticity, a one-way ANOVA revealed a significant effect, *F*(2,124) = 7.62, *p* = 0.001, η^2^ = 0.11 (see Figure 1)^4^. The results of *a priori* contrasts between the conditions were consistent with our primary hypothesis; participants in the Switching condition (*M* = 4.37, *SD* = 1.17) reported feeling significantly less authentic than those in the No Switching condition (*M* = 4.99, *SD* = 1.23), *t*(124) = 2.54, *p* = 0.01, *d* = 0.52, and the Control condition (*M* = 5.29, *SD* = 0.94), *t*(124) = 3.83, *p* < 0.001, *d* = 0.87. The No Switching and Control conditions did not significantly differ on authenticity, *t*(124) = 1.21, *p* = 0.23, *d* = 0.27.

To test the downstream effects of frame switching on well-being via authenticity, we conducted simple mediation analyses with ordinary least squares using Hayes’ PROCESS macro in SPSS (Hayes, 2012), following procedures for models with a multicategorical independent variable as outlined in Hayes and Preacher (2014). Conditions were dummy coded to specify the Switching condition as the reference group, resulting in two contrasts: (1) Switching vs. No Switching, and (2) Switching vs. Control^5^. Our original model amalgamated life satisfaction and social approval items into a single well-being outcome variable. After finding no significant indirect effects with this model, however, we conducted further exploratory mediation analyses on separate models for life satisfaction and social approval^6^. These analyses revealed that frame switching indirectly negatively influenced life satisfaction through its negative effect on authenticity but did not indirectly affect social approval.

**FIGURE 2:**
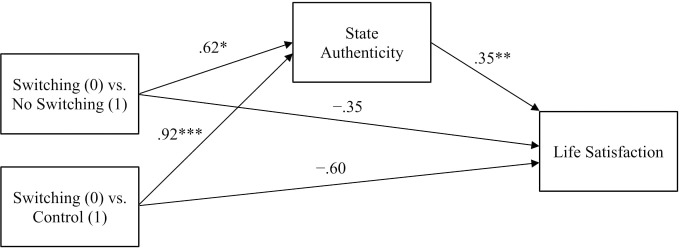
Study 1 mediation model showing the effects of frame switching on life satisfaction via state authenticity with relative direct effects of condition on life satisfaction, ^∗^*p* < 0.05, ^∗∗^*p* < 0.01, ^∗∗∗^*p* < 0.001.

In the life satisfaction model (see Figure 2), consistent with the ANOVA results, participants in the Switching condition reported having felt less authentic compared to those in the No Switching (*a*_1_ = -0.62) and Control (*a*_2_ = -0.92) conditions. Second, authenticity positively predicted participants’ life satisfaction, *b* = 0.35, *p* < 0.01^7^. Thus, when participants remembered feeling less authentic during the recalled event, they felt less satisfied with their life currently. Supporting our prediction, bias-corrected bootstrap (10,000 samples) confidence intervals for the indirect effects were below zero, indicating that frame switching significantly decreased life satisfaction by negatively affecting authenticity. Switching had negative indirect effects on life satisfaction via authenticity compared to No Switching (*a*_1_*b* = -0.22, [95% CI: -0.53, -0.04]) and to Control (*a*_2_*b* = -0.32, [CI: -0.63, -0.11]).

### Discussion

As predicted, the results from Study 1 show that frame switching decreases state authenticity and indirectly decreases life satisfaction via reduced state authenticity. Specifically, when biculturals reflect on a time when they adapted their behavior to fit with one of their cultures, they also recall having felt less authentic. This decrease in authenticity held whether frame switching was compared to actively not switching, where biculturals’ behavior did not change in response to cultural context, or an instance of mundane switching, where biculturals’ behavior changed in response to the time of day^8^. Further, the mediation results suggest that the consequences of frame switching may go beyond authenticity, having downstream repercussions for biculturals’ well-being in terms of life satisfaction.

The results of this study point to the complexity of the advantages and disadvantages of frame switching. An interesting implication of the current findings is that biculturals may willingly accept certain consequences of frame switching as a necessary sacrifice in order to fulfill their relationship and belongingness needs. Although frame switching can make them feel less authentic and lower their personal well-being, biculturals may feel that the relational well-being gained by maintaining their connection to and acceptance by each of their cultural groups outweighs their sacrifices. However, the results showed that whereas switching made biculturals feel less authentic and subsequently less generally satisfied with their lives, it did not directly or indirectly affect their impressions of social approval. One possible explanation for the latter null finding is that frame switching in a Western context has two opposing effects on social approval. On the one hand, the purpose of frame switching may well be to gain or maintain social approval by fitting in with each culture. Thus, when biculturals are focusing on their successful fitting in with others, they may anticipate that others approve of them more when they are switching. On the other hand, the inconsistency necessitated by frame switching is likely met with social disapproval in the mainstream culture. Thus, when biculturals are focusing on their behavioral inconsistency, they may realize that others might disapprove of them when they are switching. These two opposing effects on social approval highlight the paradox of frame switching in Western societies: biculturals’ attempts to gain acceptance from both of their cultures despite personal costs can actually undermine their chances of acceptance from one of their cultures.

The findings of Study 1 provide some initial evidence that frame switching can come at a cost to biculturals, particularly when their behavioral inconsistency is made salient within a dominant cultural context that associates inconsistency with inauthenticity. Biculturals living in Western societies may compromise their sense of authenticity and personal aspects of their well-being in their attempts to fit in with both of their cultures.

## Study 2

Study 1 demonstrated that frame switching can negatively impact the way biculturals see themselves, highlighting potential intrapersonal consequences. Biculturals may readily bear these consequences in exchange for the interpersonal gains of being accepted by each of their cultures. Ironically, however, these sacrifices may be made for naught when members of certain cultures ultimately disapprove of biculturals’ inconsistent behavior. In Study 2, we explore possible social consequences of frame switching in a Western context. Mainstream members of these societies—typically White monoculturals of Western European cultural heritage—may be even more likely than biculturals to have strongly internalized their culture’s values and expectations regarding behavioral consistency and its ties to authenticity. Thus, mainstream individuals may be especially likely to react negatively to others’ frame switching, forming less favorable impressions of biculturals who do so. Consistent with the way biculturals saw themselves in Study 1, we predicted that mainstream participants in this next study would judge a bicultural to be less authentic if he frame switches than if he does not. Further, the damaging effect of switching on authenticity would lead participants to also evaluate the bicultural less positively on fundamental trait dimensions.

### Methods

#### Participants

One hundred and sixteen mainstream Canadian undergraduates completed the study online for course credit. Eligibility criteria were that participants were White and had only White parents, were born in Canada, and had parents born in the United States, Canada, or Western Europe excluding Southern Europe^9^ (e.g., Italy, Portugal, Greece; Lalonde et al., 2013). Prior to any data analysis, we excluded participants who failed more than one of four attention checks (e.g., recall the name and cultures of the bicultural in the vignette) or indicated that they did not complete the study honestly and attentively (*n* = 19). These exclusions resulted in a final sample of 97 participants^10^ (66 females, *M*_age_ = 20.73, *SD*_age_ = 4.45).

#### Design and Procedure

After providing informed consent, participants were randomly assigned to read a vignette about a bicultural in one of three conditions: (1) Switching (*n* = 38), where the bicultural’s behavior differs depending on which cultural group he is with, (2) No Switching Control (*n* = 30), where the bicultural’s behavior is the same regardless of which cultural group he is with, or (3) Neutral Control (*n* = 29), where no information is given about how the bicultural behaves with his cultural groups. After the manipulation, participants rated the bicultural’s authenticity and then rated his likeability, trustworthiness, warmth, and competence^11^. Finally, they completed demographic measures and were debriefed.

#### Materials

##### Bicultural vignette

All participants read a short vignette about Miguel Wong, a Canadian-born Mexican Chinese bicultural. We chose Mexican and Chinese because we believed that mainstream Canadian participants would be familiar with these cultures while also perceiving them to be distinct. Both cultures also represent out-groups for participants, which should isolate the intended effects of frame switching from any potential confounding effects of in-group bias that may have arisen if the target had been of mixed White heritage. The three vignettes start with the same basic information about Miguel:

“Miguel Wong is a 27-year-old graduate student completing a Master’s degree in Kinesiology. He is passionate about health and exercise and plans to have a career related to these interests. Miguel’s hobbies include playing sports, reading, and cooking. Miguel is Canadian, and his cultural background is Chinese on his father’s side and Mexican on his mother’s side. He identifies with both his Chinese and Mexican cultural heritage, and he regularly spends time with members of each culture, including friends, family, and coworkers.”

The next part of the vignette differed by condition. The Switching condition read:

“Miguel behaves differently depending on which cultural group he is with, so his behavior is more typically Chinese when he is with Chinese people, and more typically Mexican when he is with Mexicans. For instance, Miguel tends to be more calm, rational, and introverted when he is with Chinese people, but he tends to be more energetic, original, and extraverted when he is with Mexicans.”

The No Switching condition read:

“Miguel doesn’t tend to behave any differently depending on which cultural group he is with, so his behavior is largely the same regardless of whether he is with Chinese people or Mexicans. For instance, Miguel tends to be consistent, tactful, and athletic when he is with Chinese people and when he is with Mexicans.”

In the Control condition, the vignette did not provide any additional information.

The traits chosen to describe Miguel’s behavior in the Switching condition were based on previous cross-cultural research showing that Chinese and Mexican groups, on average, differ on extraversion and openness to experience (McCrae and Terracciano, 2005; Schmitt et al., 2007). In the No Switching condition, traits were not necessarily tied to one culture more than the other culture; they also fit with other aspects of Miguel’s description (e.g., interest in exercise and sports). Before finalizing the vignettes, we pretested a list of potential traits: 10 for behaviors more typically shown in Mexican groups (e.g., outgoing, energetic, creative), 10 for Chinese (e.g., reserved, calm, traditional), and 10 neutral (e.g., active, consistent, motivated). In a pre-test, 46 mainstream Canadian undergraduates rated the desirability of each of the 30 traits, and the final traits were selected so that there were no differences in desirability by trait-category (Mexican vs. Chinese vs. neutral, all *p*s > 0.48) or by condition (Switching vs. No Switching, *p* = 0.50). The pre-test ensured that any effects of the vignettes were driven by whether Miguel frame switches or not rather than by the desirability of the set of characteristics he manifests in each condition.

##### uthenticity

English and Chen’s (2011) 4-item measure of subjective authenticity (adapted from Shelton et al., 2005) was reworded in order to assess impressions of a target’s authenticity rather than one’s own authenticity. We replaced one item from the English and Chen (2011) measure that would have stated “Miguel changes himself to get along with others” because we believed this to be too explicitly tied to the content of the Switching and No Switching vignettes, thus resembling a manipulation check more than a measure of impressions of authenticity. This item was replaced with a created item asking for a global assessment of perceived authenticity: “Overall, I think Miguel is an authentic person.” The other three items (α = 0.89) were “Miguel is being himself with others,” “Miguel is artificial in his interactions with others” (reverse-scored), and “Miguel expresses his true attitudes and feelings during his interactions with others,” rated on 7-point scales from 1 (*strongly disagree*) to 7 (*strongly agree*).

##### Trait evaluations

*Likeability*. To gauge Miguel’s likeability, participants responded to nine items (α = 0.87) on 7-point ratings (1: *strongly disagree* to 7: *strongly agree*, Cila and Lalonde, 2015). Example items include “If I met Miguel, I think I might get along with him,” “Miguel seems like a person I would try to avoid” (reverse-scored), and “Overall, I think Miguel is a likeable person.”

*Trustworthiness*. We created a single item on impressions of Miguel’s trustworthiness, “Overall, I think Miguel is a trustworthy person,” rated on a 7-point scale from 1 (*strongly disagree*) to 7 (*strongly agree*).

*Warmth and competence*. Participants also rated how warm (e.g., “friendly,” “good-natured”; α = 0.86) and how competent (e.g., “skillful,” “independent”; α = 0.85) they perceived Miguel to be, using 13 items from previous measures (Fiske et al., 2002; Cuddy et al., 2007, 2008). Responses were recorded on 5-point scales from 1 (*not at all*) to 5 (*extremely*).

### Results

**FIGURE 3:**
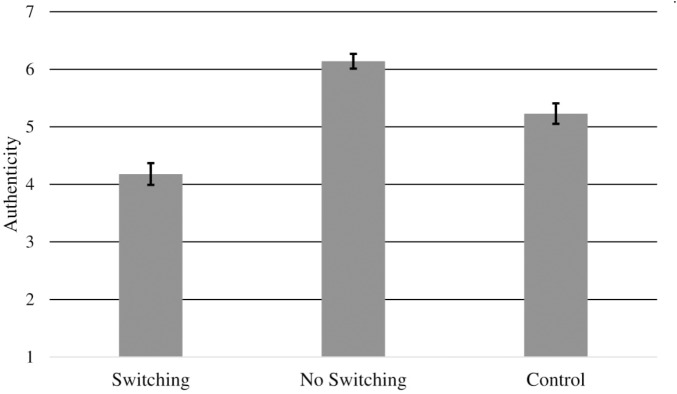
Study 2: Average authenticity ratings (±*SE*) by condition.

Testing our first hypothesis about the effect of condition on authenticity, a one-way ANOVA revealed that authenticity ratings differed significantly across condition, *F*(2,94) = 33.85, *p* < 0.001, η^2^ = 0.42 (see Figure 3). As hypothesized, participants believed that Miguel was less authentic in the Switching condition (*M* = 4.18, *SD* = 1.16) compared to the No Switching condition (*M* = 6.14, *SD* = 0.70), *t*(94) = 8.18, *p* < 0.001, *d* = 2.04, and to the Control condition (*M* = 5.23, *SD* = 0.96), *t*(94) = 4.34, *p* < 0.001, *d* = 0.98. Unexpectedly, the No Switching condition increased authenticity compared to Control, *t*(94) = 3.56, *p* = 0.001, *d* = 1.08.

**FIGURE 4:**
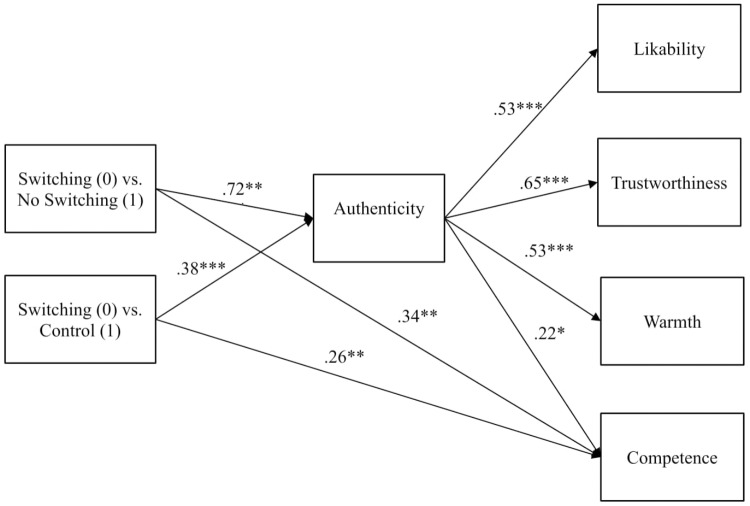
Study 2 multivariate mediation model showing the effects of frame switching on trait evaluations via authenticity with relative direct effects of condition on competence, ^∗^*p* < 0.05, ^∗∗^*p* < 0.01, ^∗∗∗^*p* < 0.001.

Given the multiple dependent measures, we built one path model in order to test the downstream effects of frame switching via authenticity simultaneously instead of conducting separate mediation analyses for each outcome. We first dummy coded the three conditions such that the Switching condition served as the reference group; the two resultant contrasts (Switching vs. No Switching, Switching vs. Control) represented the two comparisons of interest and were thus specified as the orthogonal predictors in this multivariate mediation model. The rest of the model included authenticity as the mediator and likeability, trustworthiness, warmth, and competence as outcomes. Tested with Mplus Version 8 (Muthén and Muthén, 1998–2017), the initial path model showed an unsatisfactory fit to the data: χ^2^(8) = 18.24, *p* = 0.020, CFI = 0.965, TLI = 0.912, RMSEA = 0.115, 90% CI [0.04, 0.19], SRMR = 0.033. Two fit indices (TLI and RMSEA) exceeded conventional thresholds for an acceptable fit (Hu and Bentler, 1999), and the significant chi-square was noteworthy due to the relatively small sample size (Kline, 2011). As suggested by correlation residuals and modification indices, one major area of the model–data discrepancies was that the direct effects of both contrasts on competence were non-zero, indicating that authenticity did not fully mediate the effects of frame switching on competence. As such, we added the two direct pathways, and the model fit became excellent: χ^2^(6) = 6.46, *p* = 0.38, CFI = 0.998, TLI = 0.995, RMSEA = 0.028, 90% CI [0.00, 0.14], SRMR = 0.049. See Figure 4 for final model.

Mirroring the ANOVA results, participants in the Switching condition rated Miguel lower on authenticity compared to those in the No Switching (*a_1_* = -0.72) and Control (*a_2_* = -0.38) conditions. Authenticity ratings significantly predicted ratings on the four other desirable traits. When participants saw Miguel as less authentic, they also saw him as less likeable (*b* = 0.53), trustworthy (*b* = 0.65), warm (*b* = 0.53), and competent (*b* = 0.22). More importantly, bias-corrected bootstrap confidence intervals with 2,000 resamples for each of the indirect effects were below zero for three of the four outcomes, indicating that frame switching significantly decreased Miguel’s rating on likeability, trustworthiness, and warmth by negatively affecting authenticity. Compared to No Switching, Switching had negative indirect effects on likeability -0.64 [95% CI: -0.90, -0.38], trustworthiness -1.14 [CI: -1.56, -0.77], and warmth -0.49 [CI: -0.70, -0.31], but not competence -0.21 [CI: -0.45, 0.06]. Compared to Control, Switching also had negative indirect effects on likeability -0.34 [95% CI: -0.56, -0.16], trustworthiness -0.61 [CI: -0.99, -0.29], and warmth -0.26 [CI: -0.44, -0.12], but not competence -0.11 [CI: -0.28, 0.02]). In sum, this model revealed that frame switching indirectly negatively influenced likeability, trustworthiness, and warmth through its negative effect on authenticity. There was no significant indirect effect of frame switching on competence via authenticity, but frame switching directly lowered competence.

### Discussion

These results generally support both of our hypotheses about the socially damaging effects of frame switching in a Western context. Mainstream Canadians rated the target bicultural as less authentic when he frame switched compared to when he actively did not frame switch and when they did not know anything about his behavior. Moreover, in both comparisons, when frame switching compromised the bicultural’s authenticity, he was subsequently seen as less likeable, trustworthy, and warm, though not less competent.

These findings have potentially impactful implications for biculturals in Western contexts. They identify a possible cultural barrier, in that mainstream members may not give allowance to biculturals’ behavioral inconsistency on account of their belonging to multiple cultures. We were surprised by the magnitude of differences between the three conditions on authenticity – each about a full point on a 7-point scale, producing a large standardized effect (Cohen J., 1988) – because our sample consisted of undergraduates at a liberal, very culturally diverse university in a Canadian city that prides itself on its multiculturalism. Before initiating this study, we were concerned that this sample may not endorse Western cultural associations between consistency and authenticity strongly enough to affect their reactions to our bicultural. The results show, however, that these mainstream Canadians did penalize frame switching in their impressions of authenticity, and this lead to less positive impressions on other desirable traits as well. Thus, the results from our sample might underestimate the effect compared to Western cities that are relatively less diverse. These downstream consequences are worth noting because they themselves could foster further social consequences for biculturals. For instance, if mainstream Westerners see frame switching biculturals as less likeable, trustworthy, and warm, these impressions may make them less likely to form close relationships with biculturals and behave less prosocially, among other consequences. On this topic, it is worth noting that although frame switching did not indirectly affect the bicultural’s competence through authenticity, it did decrease impressions of competence on its own. This effect may come with its own host of penalties for biculturals living in Western societies because being seen as less competent by members of the power-holding mainstream culture may cost frame switching biculturals opportunities in their education, career, etc. However, testing any of these suggested downstream consequences of frame switching require future studies where participants directly interact with biculturals rather than judging them from a third-person standpoint, as was the case in this initial investigation.

An especially illuminating result was that the negative effects on authenticity and other traits held when frame switching was compared to a control condition that did not give participants information about the biculturals’ behavior, instead providing only basic information that included his cultural background. If there was no difference between the switching and control conditions, and participants had penalized the bicultural in both compared to the no switching condition, we might have inferred a general bias toward the bicultural that was alleviated by adhering to the mainstream cultural preference for consistent behavior. The results show, in contrast, that impressions of the bicultural with no behavioral information were mildly positive, and that frame switching cost him his authenticity and other desirable traits. This implies that mainstream Canadians’ negative reactions were driven by the bicultural’s frame switching rather than by simply any bias they might have toward his particular minority cultures or toward his bicultural status in general. Further, the unexpected finding that actively not switching boosted the bicultural’s authenticity strengthens our assertion that mainstream Westerners value and reward behavioral consistency, which is fundamentally at odds with the act of frame switching.

## General Discussion

The present research unveils psychological and social consequences of frame switching for biculturals. Western philosophical traditions and lay theories create a normative cultural framework in which behavioral inconsistency is equated with inauthenticity, inherently setting up frame switching biculturals for a fall. When biculturals frame switch, their main goal may be to achieve cultural fit with both of their cultures by matching themselves to each one at a time, without permanently sacrificing their fit with one for the other. Paradoxically, for biculturals in Western contexts, this way of maintaining cultural fit creates a fundamental misfit with the mainstream culture’s beliefs and expectations, as the inconsistency of their behavior while frame switching makes them see themselves, and makes others see them, as less authentic which can have downstream consequences. Thus, despite frame switching’s benefits of increasing cultural fit within each frame (e.g., LaFromboise et al., 1993; Hong et al., 2000), we show that the act of switching between frames can be costly in certain cultural contexts.

### Complexifying Cultural Fit

This research takes a novel approach to examining cultural fit by considering it as an active process, asserting that the way people attempt to fit with their cultures may be as important to consider as their overall levels of cultural fit. In the case of biculturals, for instance, a more traditional focus may have been to examine the outcomes associated with the overall amount of overlap between a bicultural with each of their two cultures (e.g., values, personality, etc.). While such an individual differences approach would likely be informative, it might provide an incomplete picture of how cultural fit affects biculturals because it neglects the fact that their level of fit with each culture changes depending on context, and that doing so interacts with the larger cultural context shaping the experience and consequences of cultural fit. By considering frame switching as a process of cultural fit, we have unearthed a set of possible negative effects of cultural fit in a Western context that may have otherwise been missed. In doing so, we not only challenge assumptions that cultural fit is always beneficial, but also reveal the potential quagmire biculturals may face when trying to fit in with both of their cultures in a Western context – frame switching to increase their fit benefits biculturals in each frame, but if their behavioral inconsistency is made salient to themselves or others, it may undermine the very thing they are trying to achieve – cultural fit. Our work, therefore, broadens the scope of cultural fit research to include the ways people achieve fit and unveils complex relationships between the advantages and disadvantages of cultural fit.

### Understanding Biculturals’ Shared Experiences

Situated within biculturalism research, the findings of these studies add to a growing body of work examining the unique products of the common processes biculturals use to negotiate their cultures (Tadmor et al., 2012; Saad et al., 2013; Zhang et al., 2017). As advocates of a transformative theory of biculturalism, we have elsewhere encouraged researchers to find the ways that biculturals are more than the sum of their parts (West et al., 2017); how do the specific ways biculturals negotiate their cultures affect their experiences and characteristics, beyond the effects of each of their cultures independently? The current studies demonstrated potential consequences of frame switching amongst a diverse array of biculturals within a shared cultural context. In Study 1, the biculturals we sampled named 38 different national cultures as those they felt most personally connected to. Despite this diversity, our results suggest that their common experiences of frame switching can have similar repercussions in a shared Western context, coming at a cost to their sense of authenticity and consequently their personal well-being. In Study 2, even though our bicultural target had a specific cultural background, the design and results of our manipulation affirm that the negative social effects were driven by mainstream Canadians’ reactions to frame switching rather than the particular cultures. Thus, these studies emphasize how the process of frame switching can uniquely affect biculturals’ experiences. To our knowledge, this is some of the first work to establish causal relationships between a specific bicultural negotiation process and psychological and social outcomes.

### Limitations and Future Directions

Though this research contributes some preliminary, novel findings to the literature on cultural fit and biculturalism, the studies presented are limited in at least the following ways. First, Study 1 relied on biculturals’ recollections of an instance of frame switching and their feelings of authenticity during the event. This method does not allow us to observe participants’ real-time experiences and so our findings may not reflect how biculturals actually feel while they are in a particular frame. However, what is interesting about these results is that biculturals’ memory may be biased toward feeling less authentic when recalling frame switching regardless of how they feel when actually doing so. This highlights a point that was made early on in this article, about the distinction between the moment-to-moment experiences of authenticity and recall about authenticity, the latter of which may be more heavily influenced by cultural expectations and beliefs. Biculturals may commit an error similar to introverts who remember feeling less authentic when acting extraverted despite actually feeling more authentic when doing so (Fleeson and Wilt, 2010). During the meta-cognitive process of retrospecting, biculturals in Study 1 may have been influenced by internalized associations between behavioral consistency and authenticity, which served as an interpretive lens for making sense of their frame switching experiences. Future experience sampling or daily diary-based studies could examine how authentic biculturals feel during moments of frame switching, to see if these states differ from what biculturals might expect to feel based on shared lay beliefs about what constitutes authentic behavior.

Another limitation concerns the cultural background of participants in relationship to the bicultural’s background in Study 2. Participants were mainstream Canadians who learned about how a bicultural behaves with his two other cultures. In this study, we intentionally chose two non-Canadian cultures for the bicultural’s background in order to avoid possible in-group signaling effects that might have biased participants’ reactions to frame switching. If the participants’ culture was one that the bicultural was described as switching between, participants may have reacted negatively because of the bicultural switching away from participants’ own culture, as would be predicted by research on prejudice and intergroup processes (Jones, 2005; Johnson and Kaiser, 2013; Johnson and Ashburn-Nardo, 2014) and evidence of cultural matching (Taylor et al., 2007; Kim et al., 2008; De Leersnyder et al., 2014; Kokkoris and Kühnen, 2014; Tsai, 2017), and not necessarily because of a preference for not-switching. Nonetheless, because in the current study participants’ culture was not one of the cultures that the bicultural was described as frame switching between and engaging with, it is unknown how intergroup processes may play a role in this phenomenon. In order to address this limitation, future research should examine the reactions of individuals who belong to one of a bicultural’s groups (e.g., minority perceivers) when they are aware versus unaware that a bicultural frame switches. Follow-up studies like this that integrate intergroup processes would allow us to model richer situations that would feasibly occur in biculturals’ lives.

A related limitation of Study 2’s method is that participants were assigned an “omniscient” role by receiving explicit information about the bicultural’s behavioral (in)consistency and then gave their impressions without directly interacting with him. A detached, third-person perspective may not reflect how people naturally form impressions of biculturals. In real life, others may be most likely to learn that a bicultural frame switches when they are interacting with a bicultural in a mixed-cultural setting where one of the bicultural’s other cultural groups are also present (e.g., a wedding, family gathering, party). Perceivers’ reactions to frame switching in situations where they are actually interacting with biculturals may differ from the more artificial scenario we created in this study. To address this issue, we intend to build on the initial observations presented here by examining more naturalistic frame switching situations to see if perceivers react differently to biculturals when interacting face-to-face.

Another consideration for both studies surrounds the issue of demand characteristics elicited by the explicit manipulation of behavioral consistency and ensuing judgments of authenticity. Although the manipulations and design of both studies likely made evident our focus on the effects of consistency on authenticity, we believe that participants’ ability to respond in the predicted ways depends on the accessibility of the cultural lay beliefs about the consistency–authenticity association. Thus, any demand characteristics were likely shaped at least as much by the Western cultural expectations that we intended to study as by participants’ desire to fulfill a “good subject” role (Orne, 1962; Nichols and Maner, 2008). It may also be worth noting that responding according to our hypotheses in both studies required participants to go against competing incentives: to protect their own self-esteem in Study 1, and to avoid appearing racially biased in a multiculturalism-promoting setting in Study 2. Nonetheless, future studies should include subtler ways of testing our hypotheses that would reduce demand characteristics that are not driven by shared lay beliefs.

A final limitation of both studies is that we have focused only on a Western context. Although our findings suggest that frame switching can have negative consequences for biculturals in the United States and Canada, we do not know how frame switching is received in other cultural contexts or by minority groups in Western contexts. Some research on culture and consistency calls into question the extent to which people from different cultures actually differ in personality consistency across roles (Church et al., 2008b, 2013; Locke et al., 2017). Similarly, authenticity may be a universally important characteristic that people gauge and value in others, and experiences of authentic states may be more similar than different across cultures (Slabu et al., 2014). However, cross-cultural differences may still exist in prescriptions surrounding what being authentic should look like (e.g., Kashima et al., 2004), as authenticity is undoubtedly a multifaceted construct with criteria that vary between people across different contexts, and these internalized guides likely color the way different people construct and interpret their own and others’ experiences. To illustrate, certain aspects of Study 1 (e.g., materials in English) may have prompted biculturals to particularly rely on Western expectations and beliefs about behavioral consistency, external influence, and authenticity when recalling how they felt while frame switching and reporting their current well-being (Zhang and Noels, 2013). It would be interesting to see if activating a different cultural frame would change the results we obtain – for instance, if eligible biculturals completed this study in Japanese, would that culture’s emphasis on dialecticism and social role fulfillment reverse our pattern of results, leading participants to recall feeling more authentic when frame switching than not? Future cultural priming studies could test this hypothesis, seeing whether different cultural frames change how biculturals interpret their frame switching experiences. Relatedly, mainstream Canadians in Study 2 presumably drew on their Western cultural understanding of authentic behavior in deeming the bicultural least authentic when he frame switched. But how might perceivers from other cultures react? If we conducted the study in East Asia, for instance, and this culture expects people to adapt their behavior, accept external influence, and fulfill social roles (as researchers have traditionally thought, e.g., Markus and Kitayama, 1991, 1998), and associates doing these things with authenticity rather than inauthenticity, then frame switching may not have the same misfit with this surrounding culture and may not evoke the same negative reactions as in Western contexts. Including conditions in future studies that emphasize other potential components of authenticity, pitting them against behavioral consistency alone, would be an insightful test of the necessity and centrality of consistency to authenticity. On the other hand, frame switching may elicit similarly unfavorable reactions for biculturals in East Asia but for reasons other than inconsistency signaling inauthenticity. Many East Asian cultures promote strong in-group/out-group boundaries and racial essentialism, and any behavior that indicates that a person has divided alliances to different groups may be construed as disloyalty^12^, especially when those other groups have clear ethnic or racial markers (Chen et al., 2018). Thus, biculturals could face similar consequences in East Asian and Western contexts but through different mechanisms. Future research with other cultural samples and in other cultural contexts is needed to determine differences and similarities in how frame switching affects biculturals’ psychologically and socially.

### Are Frames Masks or Faces?

In general, these studies suggest that frame switching could come at a cost to biculturals’ authenticity in Western cultural contexts. Whereas this may have been expected in Study 2, in which mainstream Canadians reacted negatively to switching, the effects on authenticity may not have been quite as foreseeable for the way biculturals feel about themselves. Previous research on role-consistency has suggested that a person can still feel authentic within roles despite reporting a certain amount of inconsistency between them (Sheldon et al., 1997) and that the association between cross-role inconsistency and authenticity differs between individuals and cultures (Cross et al., 2003; Kashima et al., 2004; Boucher, 2011). Therefore, biculturals may differ as to whether their cultural frames feel like masks, that inauthentically obscure the self, or like faces, that authentically express the self. Based on this, in Study 1 we explored whether the negative effects of switching on authenticity would be moderated by biculturals’ cultural identity structures (see footnotes 3 and 8). Though these results did not support this prediction, it remains possible that biculturals vary in the extent to which frame switching makes them feel less authentic and in the circumstances that evoke this effect. It may be that being in a certain cultural frame feels more authentic to biculturals than being in another, or that being in certain social roles within each frame (e.g., friend, son/daughter) may feel more or less authentic. Feelings of authenticity may also depend on the motivational nature of biculturals’ frame switching, as accepting external influence may not undermine authenticity if doing so feels self-directed and self-expressive (i.e., in line with truly held preferences and values) rather than driven solely by external pressures (i.e., seeking reward and avoiding punishment; Kernis and Goldman, 2006).

As to the process of switching itself and the inconsistency it necessitates, negative effects on authenticity may depend on the degree to which biculturals have internalized and endorse Western cultural associations between consistency and authenticity. Even biculturals who generally feel authentic within each of their cultural frames may interpret their inconsistent behavior between contexts as a sign of their own inauthenticity when their frame switching is brought to their attention in Western societies. This suggests that biculturals may not necessarily feel less authentic in the moment when frame switching unless they reflect on the inconsistencies involved. Thus, we encourage further research into the situational and individual factors that influence biculturals’ experiences of frame switching, affecting whether the switching process ultimately feels like changing masks or faces.

### To Switch or Not to Switch?

The results of these two studies are particularly relevant in our increasingly diverse Western societies, as they identify a potential source and multiple consequences of intercultural barriers. As we have argued, frame switching seems fundamentally at odds with Western cultural prescriptions that associate consistency and authenticity. Additionally, frame switching between cultures is likely an unfamiliar phenomenon to mainstream monoculturals, and this unfamiliarity may exacerbate their negative reactions to the inherent violation of their culture’s idealized expectations and beliefs. As such, learning that a bicultural frame switches may be difficult for mainstream Westerners to understand and accommodate, and the knee-jerk reaction may be disapproval, suspicion, and distance. Study 2 showed that mainstream Canadians—even in a highly liberal, diverse, multicultural context—deemed a frame switching bicultural to be less authentic, and this had subsequent consequences for likeability, trust, and warmth. In the real world, it is possible that the downstream implications could go beyond impressions. For instance, if mainstream Americans and Canadians dislike and distrust a frame switching bicultural, they may act less prosocially toward them, afford them less opportunities in society, and be less open to intimate, meaningful social or romantic relationships.

Despite these hypothetical implications for biculturals, the worst of these consequences may be restricted to contexts in which authenticity is highly valued and consistency is strongly associated with authenticity. Research within psychology and from other social sciences contests the necessity and centrality of consistency to evaluations of authenticity and suggests that this varies by context *within* as well as between cultures. A campaigning politician, for example, may face harsh fallout for endorsing different values more strongly to one cultural group of voters than another. Former United States president Obama, for instance, drew media attention by behaving differently with Black versus White people, sparking controversial reactions from Americans who questioned his authenticity and claim to each of his cultural identities (e.g., McWhorter, 2016; Timm, 2016). An international businessperson, in contrast, is less likely to be scorned (and in fact, may be praised as savvy) for adapting her pitch to fit the cultural norms of investors in one country versus another. In fact, research and training in the business world often highlights cross-cultural competency by adapting one’s behavior to contextual demands as an essential skill for leadership and success (Earley and Mosakowski, 2004; Johnson et al., 2006; Adair et al., 2007; Ang et al., 2011). Hence, frame switching does not necessarily doom biculturals in the eyes of mainstream Westerners and may potentially have positive social effects in certain circumstances. Future studies should uncover the domains within Western culture that differ in terms of emphasizing consistency and authenticity, as this may identify the boundaries of frame switching’s negative effects.

Further hope for biculturals may come in the form of intervention studies aimed at weakening mainstream Americans’ and Canadians’ associations between consistency and authenticity, or by increasing their familiarity and understanding of biculturals’ frame switching. Empirical evidence, and common knowledge, makes it clear that even the most monocultural Westerner behaves somewhat inconsistently in response to situational demands (e.g., expressing personality traits differently across social roles; Sheldon et al., 1997), and doing so is often acceptable and even expected (Nelson, 1981; Cialdini et al., 1991; Locke et al., 2017). Thus, Westerners are capable of approving, or at least not disapproving, of behavioral inconsistency across contexts. In future studies, we plan to capitalize on familiar forms of behavioral adaptation across social roles (e.g., with a boss versus with a partner) in order to coax mainstream Americans and Canadians into relating to biculturals’ frame switching experiences, hopefully mitigating the negative effects found in the present studies.

## Conclusion

Biculturals face the complicated task of trying to fit with multiple cultures. The major implication of the current research is that the way biculturals go about doing this can affect them psychologically and socially. When biculturals frame switch, adapting themselves to each of their cultures, the inconsistency of their behavior violates Western expectations and, within this cultural context, has consequences for biculturals’ authenticity in terms of how they see themselves and are seen by others. While frame switching is undoubtedly a valuable skill for biculturals, and its benefits surely outweigh its potential costs, our work unveils the complex and sometimes paradoxical effects of frame switching, shedding light on challenges biculturals face as they go about negotiating their complex cultural worlds.

## Ethics Statement

The present studies’ protocols were reviewed and approved by the Research Ethics Committee of York University, conforming to the Tri-Council Policy Statement on Ethical Conduct for Research Involving Humans. All subjects gave written informed consent in accordance with the Declaration of Helsinki.

## Author Contributions

AW, RZ, MY, and JS contributed to the conception and design of Study 1. AW and JS contributed to the conception and design of Study 2 and performed the data collection for both studies. AW analyzed the results of Study 1. AW and RZ analyzed the results of Study 2. AW was the lead writer on the initial and subsequent drafts of the manuscript. All authors contributed to manuscript revisions.

## Conflict of Interest Statement

The authors declare that the research was conducted in the absence of any commercial or financial relationships that could be construed as a potential conflict of interest.

## Footnotes

1 Prolific’s multicultural prescreen item asks “Some researchers are interested in researching the experiences of monocultural individuals (that is, people who grew up mostly in one culture). Other researchers are interested in exploring the experiences of multicultural individuals (that is, people who are members of, or have a lineage from, more than one cultural group). Do you identify yourself as a monocultural or multicultural individual?”

2 Focusing on our primary hypothesized effect of frame switching on authenticity, we conducted a power analysis for a one-way ANOVA with three conditions. With α = 0.05 at 80% power, we needed a sample of *N* = 159 to detect a medium effect.

3 Participants also completed the Multicultural Identity Integration Scale (Yampolsky et al., 2016) prior to the recall manipulation. This measure was included because we hypothesized that participants’ cultural identity configurations (i.e., integration, compartmentalization, categorization) might moderate the effects of frame switching on authenticity. However, the results did not support this prediction.

4 To address the concern that the Accepting External Influence factor may be implicitly tied to the switching condition’s task instructions, thus functioning as a manipulation check rather than an outcome, we ran the analyses with and without this factor. The results are not contingent on the inclusion of the Accepting External Influence factor of state authenticity. When this factor is excluded, the overall effect of condition remains significant, *p* = 0.017, and the switching condition remains significantly lower on authenticity compared to the no switching condition, *p* = 0.014, and to the control condition, *p* < 0.001. The indirect effects of frame switching on life satisfaction via authenticity also remain significant for the switching vs. no switching contrast (95% CI: 0.05, 0.63) and the switching vs. control contrast (95% CI: 0.05, 0.58).

5 Though the contrasts between conditions had to be coded with the switching condition as 0 to specify it as the reference group (for indicator coding), we explain the results here and in Study 2 in terms of switching’s negative effects and therefore present the data as if switching had been coded as 1.

6 The two social approval items were significantly correlated with each of the five life satisfaction items, *r*s > 0.35, *p*s < 0.001, except for the “…live up to expectations of people…” and “…I would change almost nothing” items. Exploring the factor structure of the seven items using via a parallel analysis and EFA using ordinary least squares estimation suggested a single factor was sufficient, RMSEA = 0.08, 90% CI [0.01, 0.13], SRMR = 0.05, therefore we emphasize the exploratory nature of the mediation model in Study 1. The direct effects of condition were not significant for either life satisfaction or social approval, *F*s < 1, *p*s > 0.65.

7 The relationship between state authenticity and life satisfaction was not moderated by condition. None of the condition contrasts by authenticity interactions were statistically significant, *b*s < | 0.47|, *p*s > 0.11.

8 Surprisingly, biculturals felt less authentic when switching regardless of how integrated, compartmentalized, or categorized their identities were. Prior work shows that biculturals’ perceptions of the relationship between their cultural identities influences how they respond to their environments in many ways (e.g., Cheng et al., 2014; Zhang et al., 2014) and are also associated with different social factors (Benet-Martínez and Haritatos, 2005; Yampolsky and Amiot, 2016) and differing levels of well-being (Yampolsky et al., 2013, 2016). We did not find evidence that identity configuration moderated frame switching’s effects, though our sample size may not have provided sufficient power to detect such an interaction. In future research, we will continue to suss out potential differences between biculturals that may affect their frame switching experiences.

9 Though in this research, we use race and ancestry as a proxy for culture to recruit mainstream participants, we posit that their hypothesized reactions are the result of Western cultural influence rather than racial or genetic influences.

10 Power analysis for a one-way ANOVA with three conditions at α = 0.05 and 80% power indicated that a sample size of *N* = 159 was needed to detect a medium effect. Despite our efforts to collect this planned N, the specificity of our inclusion criteria greatly restricted the eligible members of our undergraduate pool. Given that our final sample fell short of our planned N, some amount of caution is necessary in interpreting these results, and replication with a larger sample would be ideal in future research.

11 We also measured participants’ preference for consistency in others (Cialdini et al., 1995) to see if individual differences in the endorsement of this Western cultural preference would moderate the negative effects of frame switching. However, responses on this measure were affected by our manipulation, with participants in the Switching and No Switching condition reporting higher preference for consistency compared to those in the Control condition, *p* = 0.03 and *p* < 0.01 respectively, but Switching and No Switching did not differ. Interestingly, this suggests that thinking about another person’s consistency or lack thereof bolsters Westerners’s personal adoption of the cultural preference for consistency.

12 We would like to thank Y-Y Hong, an expert on cultural frame switching, for this thoughtful suggestion.
